# The impact of fossil data on annelid phylogeny inferred from discrete morphological characters

**DOI:** 10.1098/rspb.2016.1378

**Published:** 2016-08-31

**Authors:** Luke A. Parry, Gregory D. Edgecombe, Danny Eibye-Jacobsen, Jakob Vinther

**Affiliations:** 1 Department of Earth Sciences, Natural History Museum, Cromwell Road, London SW7 5BD, UK; 2 University of Bristol, Bristol Life Sciences Building, 24 Tyndall Avenue, Bristol BS8 1TH, UK; 3 Zoological Museum, Natural History Museum of Denmark, University of Copenhagen, Copenhagen, Denmark

**Keywords:** morphological phylogenetics, Annelida, fossils

## Abstract

As a result of their plastic body plan, the relationships of the annelid worms and even the taxonomic makeup of the phylum have long been contentious. Morphological cladistic analyses have typically recovered a monophyletic Polychaeta, with the simple-bodied forms assigned to an early-diverging clade or grade. This is in stark contrast to molecular trees, in which polychaetes are paraphyletic and include clitellates, echiurans and sipunculans. Cambrian stem group annelid body fossils are complex-bodied polychaetes that possess well-developed parapodia and paired head appendages (palps), suggesting that the root of annelids is misplaced in morphological trees. We present a reinvestigation of the morphology of key fossil taxa and include them in a comprehensive phylogenetic analysis of annelids. Analyses using probabilistic methods and both equal- and implied-weights parsimony recover paraphyletic polychaetes and support the conclusion that echiurans and clitellates are derived polychaetes. Morphological trees including fossils depict two main clades of crown-group annelids that are similar, but not identical, to Errantia and Sedentaria, the fundamental groupings in transcriptomic analyses. Removing fossils yields trees that are often less resolved and/or root the tree in greater conflict with molecular topologies. While there are many topological similarities between the analyses herein and recent phylogenomic hypotheses, differences include the exclusion of Sipuncula from Annelida and the taxa forming the deepest crown-group divergences.

## 1. Introduction

Rouse & Fauchald [1] introduced many key concepts to polychaete systematics in the first comprehensive cladistic analysis of annelids. Their tree supported polychaete monophyly and established three major groupings within Polychaeta: Scolecida, Canalipalpata and Aciculata. In this scheme, palps were an important synapomorphy of Palpata, a clade comprising Aciculata and Canalipalpata, which excluded the more simple-bodied scolecids. While Aciculata and Canalipalpata and their respective subclades were supported by numerous synapomorphies, scolecids were united by absences, and it has long been suspected that they are an artificial group united by multiple independent losses [2]. Early molecular studies of Annelida found little resolution and failed to resolve many polychaete higher taxa recognized by morphologists as monophyletic [3]. However, these analyses clearly indicated that clitellates and echiurans, and possibly sipunculans (the latter two traditionally separated as distinct phyla), are derived subgroups of polychaetes [4–10]. The application of phylogenomics to annelids has begun to recover topologies that are more congruent with morphological scenarios. Support has emerged for the monophyly of two main polychaete clades. The first is composed of errant polychaetes and is similar in composition to the clade Aciculata [1]. Early transcriptomic analyses recovered a monophyletic group containing all the aciculate taxa considered plus Orbiniidae [11], but later analyses have instead recovered monophyletic groups composed of Sipuncula + Amphinomidae and Phyllodocida + Eunicida, with Orbiniidae nested within a clade of sedentary polychaetes [12,13].

The clade of sedentary polychaetes includes many of the ‘Scolecida’ together with clitellates, echiurans and many of the taxa originally in ‘Canalipalpata’ such as serpulids and sabellids, cirratuliforms, terebelliforms and siboglinids [11,12].

Early classifications of annelids considered the interstitial ‘archiannelids' to be an early-diverging clade primarily owing to their small body size and morphological simplicity [14]. It has since been recognized that the supposedly primitive characters among archiannelids are in fact adaptations to the interstitium [15] and the assemblage is not a natural grouping [16,17]. A polyphyletic ‘Archiannelida’ is also supported by molecular data, which suggest that an interstitial lifestyle has evolved numerous times within annelids [13,18].

Phylogenomic analyses have recovered a rather heterogeneous assemblage of polychaete families forming the deepest divergences of the annelid tree [12,18]. These early-branching taxa include Magelonidae, Oweniidae, Chaetopteridae, Amphinomidae, Sipuncula [12] and Lobatocerebridae, as well as Myzostomidae in some analyses [11,13]. These families present unusual and disparate morphologies, and consequently it is unclear what they contribute to our understanding of primitive characters for the phylum. This is represented in the uncertainty in crown node ancestral state reconstructions for key characters, such as the morphology of the palps or the presence or absence of aciculae [12,19]. Incongruence between morphological and molecular phylogenies has previously been discussed as a rooting issue [3,20], and numerous placements of the root of the annelid tree have been proposed and discussed based on morphological, functional and palaeontological grounds [2,21–23]. The origin of segmentation has featured heavily in discussions of the position of the annelid root. Key competing hypotheses have either advocated a clitellate-like ancestor and monophyletic Polychaeta, with segmentation evolving to compartmentalize the coelom for hydrostatic burrowing [22], or a placement of clitellates within the polychaetes, with the evolution of parapodia and chaetae forming a key step in the origin of segmentation [16,21]. Positioning the annelid root within the polychaetes is also supported by a literal reading of the fossil record, with polychaetes first appearing in the early Cambrian [24–26], echiurans possibly in the Carboniferous [27] and clitellates first represented by leech cocoons in the Triassic [28,29].

Palaeontologists and other evolutionary biologists have long recognized the importance of fossils for inferring phylogenies based on morphological data [30,31], as they are more likely to provide direct evidence of ancestral morphologies that can be crucial in polarizing morphological characters and identifying homoplastic characters. In spite of this, studies that integrate the palaeontological record into studies of annelid phylogeny have lagged behind the pace of results using molecular sequence data. Previous cladistic analyses that have incorporated annelid fossils have focused either on single exemplary fossils from individual localities [32,33] or numerous fossils from single localities [34]. Such analyses have made use of the matrix of Rouse & Fauchald [1] or a slightly modified version of that matrix. Results have been mixed, typically resolving a tree identical to that of Rouse & Fauchald [1], with fossils recovered as primitive members of major clades [32] or in suspect clades containing only fossils with no clear synapomorphies [34]. Analyses aimed at addressing the position of Cambrian taxa have either used small numbers of characters and terminals coded at suprafamilial taxonomic rank, some of which are of dubious monophyly, like ‘Scolecida’ [35,36], or have offered poor resolution for the taxon of interest [24].

Cambrian taxa are in a critical position in discussions of early annelid evolution as they may represent primitive and unusual morphologies [37], and are not readily assigned to any extant higher annelid taxon [36,37]. Early fossils have long been regarded as key sources of phylogenetic information for reconstructing phylogeny from morphological data [31], and a recent study of arthropod phylogeny suggested that inclusion of fossil data improves congruence of morphological and molecular trees for deep phylogenetic questions [38]. Consequently, we aim to explore the effects of including fossil data in cladistic analyses of annelids.

We present analyses of 80 taxa and 192 morphological characters, including a sample of 62 extant annelids, five outgroups from within Lophotrochozoa and 16 Palaeozoic fossil terminals. Fossil taxa include polychaetes, sipunculans and the ‘halwaxiids’, the latter a problematic (and probably non-monophyletic) assemblage of lophotrochozoan fossils that have been interpreted as stem and/or crown-group representatives of brachiopods, molluscs and annelids [39,40]. Extant taxa include those resolved at the base of the tree in phylogenomic analyses [12], namely Oweniidae, Magelonidae, Chaetopteridae and Sipuncula, including the Cambrian fossil sipunculans described by Huang *et al*. [41]. Five interstitial polychaete taxa were included (*Mesonerilla, Protodrilus, Saccocirrus, Protodriloides, Polygordius*).

**Figure 1.**
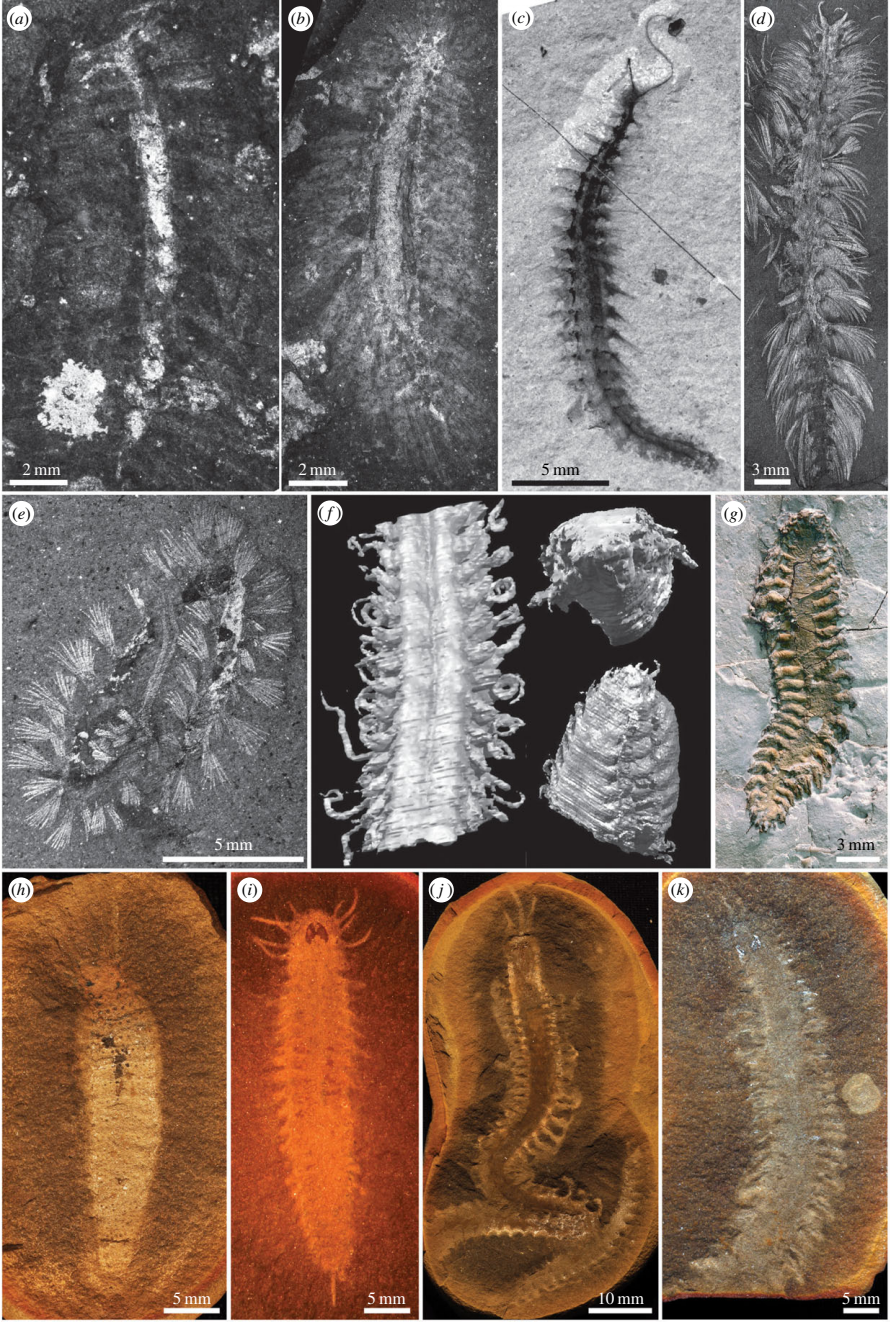
Fossil annelids used in this study. (*a*) *Pygocirrus butyricampum* MGUH31365; (*b*) *Phragmochaeta canicularis* MGUH3088; (*c*) ROM62927, undescribed polychaete from Marble Canyon; (*d*) *Canadia spinosa* USNM83929c; (*e*) *Burgessochaeta setigera* USNM198705; (*f*) *Kenostrychus clementsi* OUM C.29544 (top right), OUM C.29543; (*g*) *Arkonips topororum* UMMP 73795; (*h*) *Mazopherusa prinosi*; (*i*) *Fossundecima konecniorum* ROM47990; (*j*) *Esconites zelus* ROM47521; (*k*) *Dryptoscolex matthiesae* ROM48542. (*a*,*b*) Early Cambrian, Sirius Passet, North Greenland; (*c–e*) Middle Cambrian, Burgess Shale, British Columbia; (*f*) Silurian (Wenlock), Herefordshire; (*g*) Middle Devonian, Arkona Shale, Hungry Hollow, Arkona, Ontario; (*h*–*k*) Carboniferous (Pennsylvanian), Mazon Creek, Illinois. Images (*f*) and (*g*) courtesy of Mark Sutton and Derek Briggs, respectively. (Online version in colour.)

Annelid fossil taxa which are included range in age from early Cambrian to Pennsylvanian and are from Konservat–Lagerstätten exhibiting a diversity of taphonomic modes, including carbonaceous compressions (figure 1*a–e*), void fills in carbonate concretions from volcaniclastic sediments (figure 1*f*), three-dimensional pyritization (figure 1*g*) and preservation within ironstone concretions (figure 1*h–k*).

## 2. Characters and character coding

Our matrix was assembled based on the published matrices of Rouse & Fauchald [1] and Zrzavý *et al*. [5]. We adopted a multistate coding following [42], so that absence of a given character appears only once, with contingent characters coded for multiple states within a given character.

Of the 192 included characters, 141 have been used previously in the analyses of Zrzavý *et al.* [5] and/or Rouse & Fauchald [1] and Rouse [43] or for ancestral state reconstructions [11], whereas the remaining characters were defined and coded from the recent literature (see the electronic supplementary material).

We adopted a different approach for coding the presence of palps and palp homologues than in previous morphological matrices. Rouse & Fauchald [1] coded the presence of palps as an absence/presence character, and the various substates of this character were themselves coded as separate absence/presence characters. In contrast, Zrzavý *et al.* [5] coded the presence and absence of palps and buccal tentacles as a single multistate character, whereas other aspects of palp morphology were treated as separate characters (such as attachment position), this coding being retained in subsequent revisions of this matrix [11,12]. Both these approaches treat buccal tentacles as palp homologues, as taxa possessing buccal tentacles are not scored as absent for palps. The buccal tentacles in Terebelliformia are not palp homologues, as they lack the distinct innervation that characterizes true palps [44,45], and there are no palp homologues in other polychaete taxa that are derived from the buccal cavity [44].

## 3. Phylogenetic methods

There is currently a debate on the most appropriate method for analysing discrete morphological characters, which has largely focused on simulations of binary character data [46–48]. Empirical studies directly comparing these methods and implementations (e.g. maximum-likelihood versus Bayesian implementation of the *mk* model) are however comparatively rare. Consequently, we analysed our data using equal weights and under implied weighting under a range of concavity constants (*k* = 3, 5 and 10), and using maximum-likelihood and Bayesian inference. The *mkv* model was appropriate for our dataset as the correction of Lewis [49] accounts for the ascertainment bias, as invariant characters were not coded during the assembly of our matrix and autapomorphies were not comprehensively coded. Parsimony analyses were performed using TNT. 1.1 [50], Bayesian analysis used MrBayes. 3.2.6 [51] and likelihood analyses used RAxML 8.2.8 [52].

Parsimony analyses used all the New Technology search options with the default options in TNT using a driven search with 1000 initial addition sequences and instructed to find the optimal topology 10 times. Support values are symmetric resampling for implied-weights analyses, and Bremer support and bootstrap replicates for equal weights. Jackknife frequencies were also calculated for equal-weights trees and are presented in electronic supplementary material, figures S1*a* and S3*a*. All resampling methods used 10 000 replicates.

Maximum-likelihood support values were generated from 1000 bootstrap replicates. Bayesian analyses were performed for 10 million generations, sampling every 1000 generations with 25% of trees discarded as burn in, resulting in a total of 7500 trees. Rate variation was modelled using a gamma distribution with four discrete gamma categories. Convergence was assessed using the average deviation of split frequencies (with convergence achieved at less than 0.01) and using Tracer 1.6, to ensure that the runs had reached stationarity prior to burn in and that all parameters had effective sample size (ESS) scores above 200. In order to assess the effects of including or excluding fossil data, all analyses were performed identically with and without fossil terminals.

The morphospace of extant annelids was explored using a principle coordinate analysis using PAST 3 [53], using Euclidean distances for the character matrix with fossil taxa excluded.

## 4. Results

**Figure 2.**
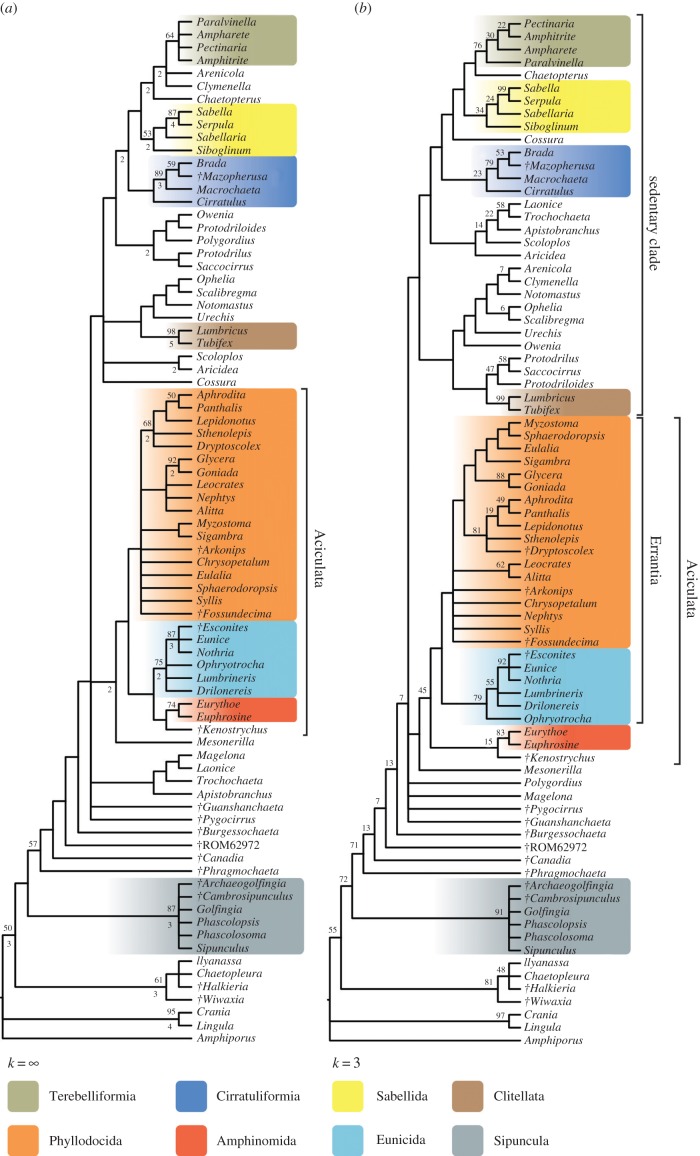
Results of parsimony analyses. (*a*) Strict consensus of 388 most parsimonious trees of length 636 under equal weighting. Numbers above and below nodes are bootstrap frequencies (less than 50% not shown) and Bremer support values, respectively. Consistency index = 0.3156, retention index = 0.6896. (*b*) Strict consensus of 328 trees under implied weighting. Numbers at nodes are present/contradicted support values expressed as frequency differences obtained from symmetric resampling. (Online version in colour.)

**Figure 3.**
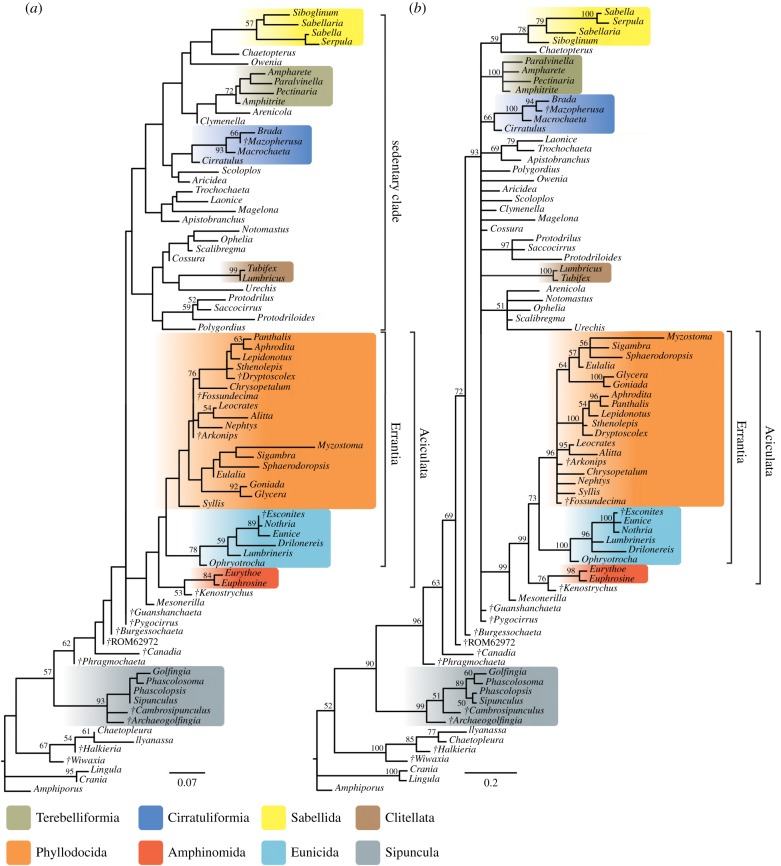
Results of probabilistic analyses using the *mkv* + *Γ* model. (*a*) Maximum-likelihood phylogram. Numbers at nodes are bootstrap frequencies from 1000 replicates. (*b*) Phylogram from Bayesian analysis. Tree shown is a majority rule consensus of 7500 trees. Numbers at nodes are posterior probabilities. Scale bar units are expected number of substitutions per site. (Online version in colour.)

The analyses including fossils (figures 2 and 3) all support the inclusion of Echiura and Clitellata within polychaetes, the polyphyly of Scolecida, and the monophyly of Aciculata. Our results support the existence of two main annelid clades, one consisting of errant polychaetes with aciculae (composed of Phyllodocida, Eunicida, Amphinomida), the other a sedentary annelid clade, which includes Echiura and Clitellata, Cirratuliformia, Terebelliformia, Sabellida (although not including Oweniidae as in [1]), and various taxa assigned to ‘Spionida’. This sedentary clade also contains the taxa that were previously classified as ‘Scolecida’, including Arenicolidae, Capitellidae, Maldanidae, Opheliidae and Scalibregmatidae. Echiurans group with either some (figures 2*a* and 3*b*) or all of these scolecidan taxa (figure 2*b*), and a clade of Opheliidae, Capitellidae and Echiura is likewise recovered from phylogenomic data [12]. Arenicolidae and Maldanidae are closely related to terebelliforms in equal-weights and likelihood analyses, a clade that is also supported by molecular data [12], although at present there are no transcriptomic data available for any maldanid taxon. The monophyly of the sedentary clade is however not resolved in equal-weights or maximum-likelihood analyses.

Sampled archiannelid taxa are neither early-branching crown-group annelids nor a clade within annelids. Our results suggest multiple independent miniaturization events within annelids, as similarly indicated by phylogenomic data [13,18] as well as previous morphological analyses [5,54]. However, the positions of the sampled ‘archiannelid’ taxa, *Mesonerilla* and a *Protodrilus*/*Protodriloides*/*Saccocirrus/Polygordius* clade, within Aciculata or the sedentary clade/grade are reversed when compared with similar clades in recent phylogenomic trees [18], although *Polygordius* is part of a basal polytomy under implied weighting and Bayesian inference. At least some members of Nerillidae possess many of the synapomorphies of errant polychaetes such as lateral antennae and parapodial cirri [2,54], and compound chaetae are present in some members of the family [2,54]. The position of the other archiannelid taxa (protodrilids and *Polygordius*) within a sedentary polychaete clade closely approximates previous cladistic analyses [43], in which they formed a clade or grade within Canalipalpata.

When fossils are excluded, the ‘traditional’ topology with Echiura and Clitellata forming successive outgroups to a monophyletic Polychaeta is recovered in a subset of the trees from parsimony with equal weights. Under implied weighting and both implementations of the *mkv* model, taxa in Sabellida and Chaetopteridae root the tree. The analyses in which fossils are excluded are highly ambiguous and poorly resolved (equal weighting; electronic supplementary material, figure S3*a*), rerooted with Sedentaria forming a grade (implied weighting, maximum likelihood; electronic supplementary material, figure S3*b–c* and S5) or both (Bayesian inference: electronic supplementary material, figure S4). In all of these analyses lacking fossils, the position of the annelid root is strongly in conflict with molecular phylogenies.

The Cambrian fossil annelids are primarily placed outside of the annelid crown group as previously proposed [35,36] and in line with the phylogenetic hypothesis outlined in [37]. In parsimony and Bayesian analyses, the Cambrian *Guanshanchaeta* and *Pygocirrus* are in a polytomy with the annelid crown group or form successive outgroups to the crown group in likelihood analyses (figure 3*a*). This further highlights the importance of pygidial cirri, a character present in these Cambrian taxa, as a synapomorphy of crown-group annelids (character optimizations shown in electronic supplementary material, figure S2) [35,36].

**Figure 4.**
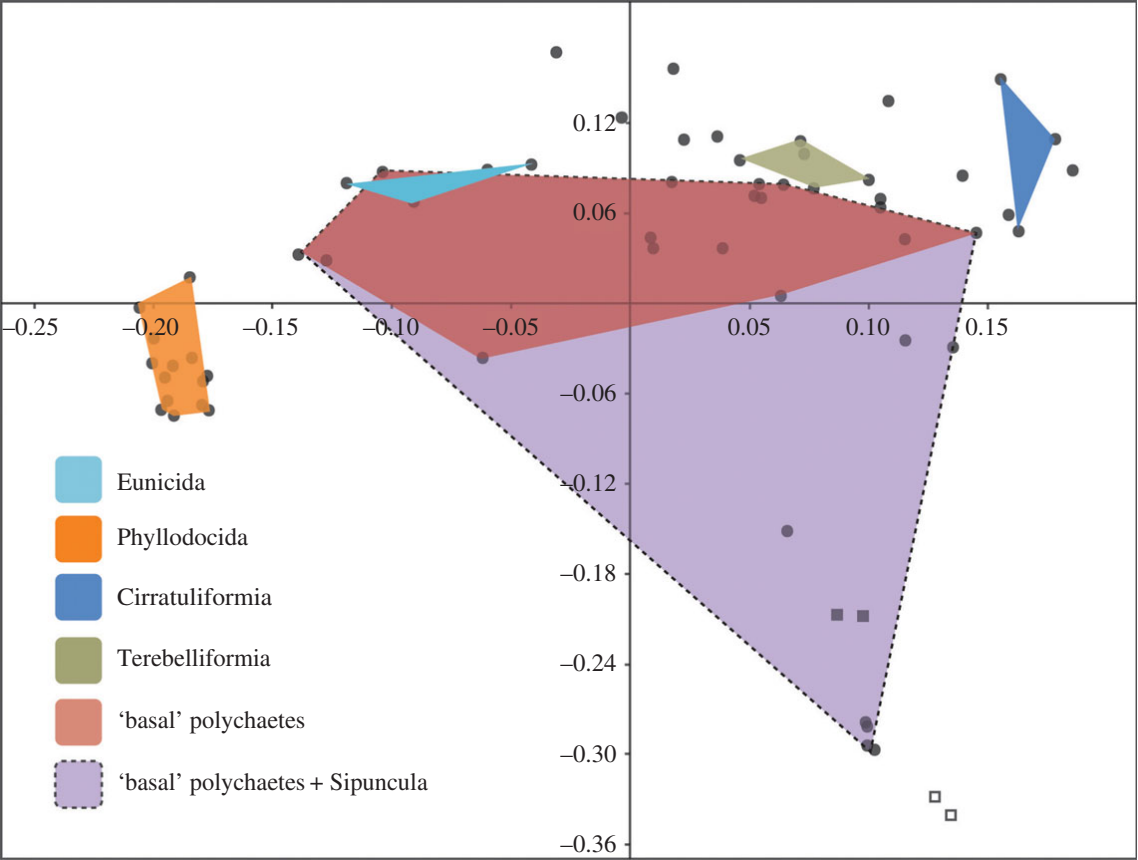
Annelid morphospace using a principal coordinate analysis derived from the cladistic dataset excluding fossils. Circles represent annelid and sipunculan taxa, open and closed squares are brachiopod and mollusc outgroup taxa, respectively. The ‘basal’ polychaetes polygon includes *Owenia, Magelona, Chaetopterus, Eurythoe* as well as the euphrosinid (the presumptive sister group of Amphinomidae). (Online version in colour.)

Our results consistently do not support the inclusion of Sipuncula within Annelida but rather a sister group relationship. This is unsurprising because the cryptic segmental characters in sipunculans [55,56] and their collagenous cuticle are annelid plesiomorphies (or secondary reductions in the case of nervous system development) and not characters derived within annelids. Consequently, based on the available data, morphological phylogenetic analyses are unlikely to include sipunculans nested within annelids. Regardless of their position within or outside annelids, sipunculans are highly autapomorphic and contribute little to our understanding of primitive characters within annelids, and they are placed far outside of annelid taxa in plots of morphospace (figure 4). While the Chengjiang taxa have previously been interpreted as crown-group sipunculans [41], Bayesian and likelihood analyses herein suggest they are members of the stem group (figure 3*a,b*). Both the fossil taxa lack a helically coiled gut, which is therefore a candidate synapomorphy of the crown group. This character is apparently reversed in a single genus of extant sipunculans (*Phascolion*), suggesting that the similarities to extant sipunculans suggested by Huang *et al.* [41] are the consequence of convergence.

Fossil taxa that are younger than the Cambrian are placed deeply nested within the annelid crown group, typically in the clades to which they were originally assigned when they were described. This suggests that the placement of the fossils is driven by phylogenetic signal in the dataset and that the placement of the Cambrian taxa is not a consequence of character loss during fossilization and ‘stemward slippage’ [57]. *Fossundecima* (figure 1*i*) from the Carboniferous is an exception, as this fossil was assigned to the Nereidae by Fitzhugh *et al.* [58], but is recovered in a polytomy at the base of Phyllodocida in our analyses (figures 2 and 3). Many of the characters observed in this fossil may be plesiomorphic for Phyllodocida or one of its subclades, such as paired lateral jaws and anterior cephalized ‘tentacular’ cirri.

Missing data in fossil taxa range from 40.6% (*Canadia spinosa*) to 63% (*Arkonips topororum*), with a mean of 50%. While missing data in fossils were previously thought to hamper phylogenetic analyses based on discrete characters, the addition of taxa that are only 50% complete can improve the accuracy of phylogenies where long branch attraction (such as the misrooting of annelid trees) hinders tree reconstruction [59]. The distribution of missing data within our trees suggests that Cambrian taxa are not recovered in the annelid stem group owing to an abundance of missing data causing them to be attracted by the root. In contrast, the Cambrian fossil polychaetes are the most complete fossil taxa in our sample, and there is a statistically significant positive correlation between fossil completeness and distance from the root to tip (electronic supplementary material, tables S1 and S2 and figure S6). While the fossil record is generally considered to decrease in quality with time, every Cambrian taxon (except *Halkieria*, which is known only from a scleritome without soft tissues) has a higher percentage character completeness than all younger fossils in the matrix, highlighting the capacity for Burgess shale-type preservation to produce uniquely complete fossils.

Key differences between our analyses and phylogenomics concern the deepest divergences. Spionidan taxa such as Magelonidae, resolved with Oweniidae as sister group to all other annelids [12], Trochochaetidae, Apistobranchidae and Spionidae are placed in a polytomy with the remainder of the crown group in equal-weights analyses (figure 2), but these taxa are nested within the sedentary clade in maximum-likelihood (figure 3*a*) and implied-weights analyses. Under implied weights, Magelonidae are recovered in a polytomy at the base of the crown group, which is similar to recent phylogenomic results in which *Magelona* and *Owenia* are the sister group of all the remaining annelids [12].

As the fossil record strongly indicates that annelids evolved from an epibenthic ancestor during the Cambrian, the inclusion of Magelonidae, Oweniidae and Chaetopteridae as a grade at the base of annelids would necessitate multiple independent origins for a sedentary lifestyle among these groups. Consequently, key characters shared with other sedentary polychaetes such as uncini would have to be considered convergent [19]. Chaeopteridae is another group resolved near the base of Annelida in phylogenomic analyses, contrary to previous morphological phylogenies, which allied it with Spionida [1]. In our trees, *Chaetopterus* is highly labile, generally allying with other sedentary taxa, although some analyses without fossils place it as sister group to all other extant annelids (electronic supplementary material, figure S3 and S5). This is not a simple consequence of *Chaetopterus* behaving as a lone ‘wildcard’ but occurs in concert with all sedentary taxa becoming unresolved as a paraphyletic grade relative to Aciculata.

Aciculata is consistently monophyletic in our analyses, regardless of the inclusion or exclusion of fossils and optimality criterion. A sister group relationship between Amphinomidae and Sipuncula has been proposed based on molecular data [12], resulting in a suspect clade with no clear synapomorphies that is strongly contradicted by our morphological data. However, as sipunculans possess no shared derived characters with any annelid subclade, any position of Sipuncula within Annelida would be similarly contradicted by morphological data. The monophyly of Aciculata is supported by numerous unique synapomorphies, including ventral sensory palps, lateral antennae and dorsal and ventral cirri (electronic supplementary material, figure S2).

We do not recover Pleistoannelida (a clade that excludes Oweniidae, Magelonidae, Chaetopteridae, Amphinomidae and Sipuncula [12]) in any of our analyses. This proposed paraphyletic early radiation of annelids is highly disparate and in our plots of annelid morphospace (figure 4) represents much of the morphological disparity of Annelida. When sipunculans are also considered, this basal radiation encompasses much of the morphological disparity of the protostome taxa included in the analysis (figure 4). Crucially, this early morphological diversity is not captured in the known Cambrian fossil record of annelids, and results of ancestral state reconstructions based on the phylogeny of extant taxa are highly uncertain, particularly for the external morphological characters observable in fossils (such as the morphology of parapodia and chaetae) [11,12].

We do, however, recover Errantia (a clade of aciculates that excludes Amphinomida) *sensu* Weigert *et al.* [12] in implied-weights, maximum-likelihood and Bayesian analyses. This group shares several characters such as compound chaetae [60] and jaws. It is not clear, however, whether the jaws of the two groups are homologous, or even whether the diverse jaws of the various taxa within Phyllodocida have a single origin.

## 5. Conclusion

Conflict between morphological and molecular trees for annelids is partly a consequence of misrooting owing to extensive secondary reduction of key characters such as parapodia, chaetae and head appendages in clitellates and echiurans. We have demonstrated that with an expanded sample of characters and fossil taxa, morphological data support the inclusion of these groups within a paraphyletic grade of polychaetes, in line with hypotheses from molecular data. While key differences persist between phylogenomic trees and the morphology-based trees presented herein, our results bolster an emerging consensus on annelid relationships and how the diversity of the extant groups was assembled. Our results suggest that the annelid ancestor was a macroscopic, epibenthic animal with paired palps and prominent parapodial lobes with numerous capillary chaetae. Secondary reduction of this complex body plan is widespread in numerous distantly related groups, which has confounded attempts to resolve annelid phylogeny using morphological data from extant taxa alone.

## Supplementary Material

Character codings and descriptions and supplementary analysis figures

## Supplementary Material

Character matrix used in analyses in NEXUS format

## Data accessibility

The datasets supporting this article have been uploaded as a nexus formatted file as part of the electronic supplementary material.
