# Supplementary material for: The impact of fossil data on annelid phylogeny inferred from discrete morphological characters
Source: Proc Biol Sci. 2016 Aug 31;283(1837):20161378. doi: 10.1098/rspb.2016.1378 (PMC5013799; doi:10.1098/rspb.2016.1378)
Supplement: Character codings and descriptions and supplementary analysis figures [file rspb20161378supp1.pdf]

Supporting supplementary material for: The impact of fossil data on annelid phylogeny inferred from discrete morphological characters

Luke Parry, Gregory D. Edgecombe, Danny Eibye-Jacobsen, Jakob Vinther

Character codings and descriptions

Characters were coded as per [1-3] and using additional characters from the matrices in [4] and [5] except where noted. Where multistate characters previously contained absence as a state, characters were recoded as multiple character complexes so that absence and presence are treated equally across all characters. Except where noted, characters are binary, with 0 denoting absence and 1 denoting presence. Cambrian fossil taxa are coded following [6-8]. New characters are indicated only where they were not considered by [1] or [3] and not where characters from previous matrices have been reformulated.

While the character matrices of Rouse and Fauchald [1] and Rouse [2] were scored at family level, taxa herein are coded at genus level. Consequently, the genera scored are those used in [3] except in cases where the presence of palp nerve roots are known from a different genus, as figured in [9, 10]. Some of the families coded in previous analyses are monotypic (e.g. Trochochaetidae, Apistobranchidae) and so the generic coding here does not deviate from previous family level analyses for these taxa.

Presence of palp nerve roots within Dorvilleidae is known only from the progenetic *Parapodrilus* [11], so consequently characters for *Ophryotrocha* were scored and the relevant nervous system characters are coded as missing where appropriate. Chaetal characters are coded specifically for *Protodriloides chaetifer* as chaetae are absent in other members of the genus [12]. Characters for sipunculan genera were coded from previous character matrices [13]. Where chracteres were mis-scored by [1] the corrections outlined in [12] and followed by [3] were followed herein. General references for the included polychate genera are listed below, with character specific references and codings outlined in the following character list.

Coding of extant annelids

Alitta [1, 3, 14-16]
Ampharete [1, 3, 9, 16]

Amphitrite [1, 3, 9, 16]

Aphrodita [1, 3, 16, 17]

Apistobranchus [3, 16]

Arenicola [1, 3, 12, 16]

Aricidea [1, 3, 12, 16]

Chaetopterus [1, 3, 12, 16]

Cirratulus [1, 3, 12, 16]

Clymenella [3, 12, 16]

Cossura [1, 3, 12, 16, 18]

Drilonereis [1, 3, 12, 16]

Dysponetus [1, 3, 12, 16]

Eulalia [1, 3, 12, 16]

Eunice [3, 16, 19, 20]

Euphrosine [1, 3, 12, 16]

Eurythoe [1, 3, 12, 16, 21]

Glycera [1, 3, 12, 16]

Goniada [1, 3, 12, 16]

Nothria [3, 16, 20, 22]

Laonice [1, 3, 12, 16]

Leocrates [1, 3, 12, 16]

Lepidonotus [1, 3, 12, 16, 23]

Lumbrineris [1, 3, 12, 16]

Macrochaeta [1, 3, 12, 16]

Magelona [3, 12, 16]

Mesonerilla [1, 3, 12, 16, 24]

Myzostoma [1, 3, 12, 16]

Nephtys [1, 3, 12, 16]

Notomastus [3, 12, 16, 25]

Nothria [3, 16, 19, 20]

Ophelia [1, 3, 12, 16]

Ophryotrocha [1, 3, 12, 16, 26]

Owenia [1, 3, 12, 16, 27]

Panthalis [1, 3, 12, 16, 23]

Paralvinella [1, 3, 9, 12, 16]

Pectinaria [1, 3, 9, 12, 16]

Polygordius [1, 3, 12, 16]

Protodriloides [3, 12, 16]

Protodrilus [3, 12, 16]

Sabella [1, 3, 12, 16]

Sabellaria [1, 3, 12, 16]

Saccocirrus [3, 12, 16]

Scalibregma [1, 3, 9, 12, 16, 28, 29]

Scoloplos [1, 3, 10, 12, 16, 30]

Serpula [1, 3, 12, 16]

Siboglinum [1, 3, 12, 16]

Sigambra [1, 3, 12, 16]

Sphaerodoropsis [1, 3, 12, 16, 31, 32]

Sthenolepis [1, 3, 12, 16]

Syllis [1, 3, 12, 16]

Trochochaeta [1, 3, 12, 16]

Character List

1. Serially repetition in nervous system. Segmental neuro-anatomy has been observed and coded as present in *Urechis* [33] and sipunculans [34]. New character.
2. Serially repeated nephridia. Serial paired nephridia are known from polychaetes, clitellates and some echiurans, including *Urechis* [35]. New character.
3. Chaetae in bundles or fascicles. Coded as present in polychaetes, brachiopods [36, 37] and *Wiwaxia* [38], absent in *Urechis* [39]. New character.
4. Serially repeated chaetae/sclerites. Coded as present in all polychaetes (except *Protodrilus*) and in *Crania* [36] and *Wiwaxia* [38]. New character.
5. Posterior growth zone. Coded as present in annelids and echiurans [40] and absent in sipunculans [41]. New character.
6. Anterior segments with cephalised cirri (equivalent to tentacular cirri in other matrices).
7. Segment 1 with notopodia onlyls
8. Anal chaetal ring. Present in *Urechis* [42]
9. Spermatozoon with acrosome. Coded as per [3], with the presence of an acrosomal tube (character 10) coded as a contingent character.
10. Spermatozoon with acrosomal tube [3]

11. Prototroch. Larval characters (11-22) follow [3], which in turn are adopted from [2, 43, 44]
12. Metatroch. Coded following [44] except for the capitellid *Notomastus,* in which a metatroch appears to be present [25]
13. Opposed band larval feeding
14. Food groove
15. Oral brush
16. Cilliated region on episphere. [3] coded both the meniscotroch and akrotroch as separate binary characters. As both these ciliary bands occur on the episphere, the presence of either is coded as a single binary character.
17. Type of cilliated region: 0 – meniscotroch; 1 – akrotroch. The akrotroch is a complete ring while the meniscotroch is ventral and anterior to the mouth [2]
18. Telotroch
19. Neurotroch
20. Apical organ/tuft
21. Annelid cross [2]
22. Molluscan cross [2]
23. Clitellum
24. Hermaphroditism
25. Sperm mitochondrial interpolation
26. Sperm flagellum: 0 – posterior; 1 – anterior; 2 – two
27. Gonoducts: 0 – nephridial/coelomoducts; 1 – separate; 2 – with distal prostate-glandular part
28. Head kidneys: 0 – protonephridia; 1 – metanephridia
29. Adult nephridia: 0 – protonephridia; 1 – metanephridia. Nephridial ultrastructual characters (30-35) are coded following [3] which were derived from [35, 45]
30. Metanephridial duct
31. Terminal cell
32. Filter of terminal cells with clefts
33. Microvillar filter
34. Nephridial podocyte lining
35. Metanephridial mantle cell

36. Distribution of segmental organs: 0 – in most segments; 1 – anterior sterile nephridia and posterior gonoducts; 2 – one pair of anterior nephridia, posterior gonoducts; 3 – restricted to a few middle segments
37. Segment 1 achaetous
38. Retractable head.
39. Palps. Any taxon with either external palps or neuroanatomical homologues [9, 10] was coded as possessing palps. Rather than coding the buccal tentacles of terebelliforms in a character complex with true palps, these structures were coded as a separate character due to substantial differences between the innervation and attachment of these structures when compared with true palps [9, 10]. Although tentacles in the buccal cavity are present in *Cossura,* they are not considered homologous [18].
40. External palps. Any taxon with paired palps or a branchial crown was coded as possessing palps, whilst many 'scolecidans' were coded as lacking external palps although neuroanatomical homologues are present, including Scalibregmatidae [9, 10].
41. Palp number: 0 – branchial crown; 1 – paired
42. Paired palp position: 0 – ventral; 1 – ventro-lateral; 2 – lateral; 3 – forming branchial crown; 4 – dorsal
43. Palp attachment: 0 – peristomial; 1 – prostomial
44. Palp nerve root 1. The presence of palp nerve roots and their fusion (characters 48-53) are coded using the table presented in [10]. New character.
45. Palp nerve root 2, new character.
46. Palp nerve root 3, new character.
47. Palp nerve root 4, new character.
48. Palp nerve root 5, new character.
49. Palp nerve root 6, new character.
50. Palp nerve root 7, new character.
51. Palp nerve root 8, new character.
52. Palp nerve root 9, new character.
53. Palp nerve root 10, new character.
54. Palp nerve root 11, new character.
55. Palp nerve root 12, new character.
56. Palp nerve roots 4 and 5 fused, new character.
57. Palp nerve roots 6 and 7 fused, new character.
58. Palp nerve roots 1, 2 and 3 fused, new character.

59. Terminal palpode
60. Buccal tentacles
61. Buccal tentacles retractable
62. Palp morphology: 0 – smooth; 1 – grooved; 2 – papillose
63. Biarticulate palps
64. Palp with internal canal
65. Median antenna. The median antenna present in *Aricidea* (Paraonidae) and *Laonice* (Spionidae) is coded as homologous with the median antenna of aciculate taxa with this character [8, 9, 12]. The median branchus of *Cossura* is attached and innervated differently and is not scored as homologous [18].
66. Lateral antennae
67. Shape of notopodia: 0 – prominent lobe; 1 – small lobes or chaetae only; 2 – notopodial ridges. Coded following [4] and [5].
68. Shape of neuropodia: 0 – prominent lobe; 1 – small lobes/chaetae only
69. Enlarged neuropodia: The neuropodia of many errant polychaetes are enlarged relative to the notopodia, presumably as an adaptation for locomotion. Exceptions include forms where the parapodia are approximately equal in size, including *Eurythoe*, *Nephtys* and *Kenostrychus.*
70. Tori
71. Notopodial tori. 71 and 72 coded using family descriptions in [12]. New character.
72. Neuropodial tori, new character.
73. Sclerites or chaetae. The sclerites of *Wiwaxia, Halkieria* and aculiferans and chaetae of brachiopods are coded as homologous with annelid chaetae.
74. Mineralised sclerites or chaetae
75. Sclerites arranged in zones, considered present in 'halwaxiids' and chitons [46, 47]. New character.
76. Sclerite distribution: 0 – dorsal and ventral; 1 – ventral only; 2 – dorsal only. *Wiwaxia, Halkieria* and brachiopods have dorsal sclerites only. The anterior ventral hooks of Echiura were coded as homologous with neurochaetae, as per Tilic, Lehrke [39]. Although Thompson and Johnson [48] considered *Esconites zelus* to have notochaetae, this is considered unlikely as although two distinct bundles are visible in some specimens,

some Eunicidae have distinct dorsal and ventral neuropodial fascicles, for example *Eunice antennata* [17]. New character.

77. Compound chaetae
78. Compound chaeta position: 0 – neuropodial; 1 – neuropodial and notopodial, coded using [14]. New character.
79. Compound chaetae with ligaments. Taxa with 'pseudocompound' chaetae coded as lacking ligaments and possessing a fold (character 81).
80. Number of ligaments
81. Compound fold. Coded for taxa with a fold or incomplete articulation in the shaft, i.e. those polychaetes described as possessing 'pseudocompound chaetae' [14].
82. Paleae
83. Tube
84. Tube building organ(s)
85. Chaetal basket, present in Flabelligeridae and *Mazopherusa* [49]. New character.
86. Capillary chaetae
87. Notopodial capillaries, coded using [14]. New character.
88. Neuropodial capillaries, coded using [14]. New character.
89. Aciculae
90. Notoaciculae, coded using family descriptions in [12]. Notoaciculae coded as present in *Nothria,* although they are absent in some other Onuphidae [22]. New character.
91. Neuroaciculae, coded using family descriptions in [12]. New character.
92. Dentate hooks, coded as present in *Tubifex* as per [50].
93. Long-handled dentate hooks
94. Uncini
95. Hooks with beard
96. Neuropodial hooks, coded using [14]. New character.
97. Notopodial hooks, coded using [14]. New character.
98. Falcate hooks
99. Neuropodial falcate hooks, coded using [14]. New character.
100. Notopodial falcate hooks, coded using [14]. New character.
101. Compound hooks, coded using [14]. New character.
102. Hooded chaetae
103. Fine silk notochaetae

104. Dorsal cirri. Coded as absent in *Sphaerodoropsis* as macro-tubercles are not homologous with these structures, although true dorsal cirri are present elsewhere in the family [31].

105. Alternating dorsal cirri

106. Lateral/dorsal cirrus organs

107. Dorsal organ

108. Ventral cirri

109. Pygidial cirri

110. Number of pygidial cirri: 0 – one pair; 1 – two or more pairs

111. Posteriorly directed dorsal chaetae. Chaetae of presumed protective function that cover the dorsum. Present in *Wiwaxia* [51], *Canadia, Phragmochaeta,* unnamed Marble Canyon polychaete (ROM62972) [6], *Aphrodita* and *Dysponetus*.

112. Complete through gut

113. Through gut shape: 0 – following anterior-posterior axis; 1 – coiled/folded; 2 – U-shaped with anterior anus

114. Gut side branches

115. Presegmental peristomium and prostomium. Coded as absent in *Canadia* and *Phragmochaeta* [6]. New character.

116. Head neuropodia. Coded as present in *Canadia* and *Phragmochaeta* [6]. New character.

117. Head notopodia. Present in *Phragmochaeta* [6]. New character.

118. Prostomium annulated cone

119. Peristomium: 0 – limited to lips; 1 – ring/rings. *Notomastus* and *Cirratulus* coded as having a ring-like peristomium and *Magelona* uncertain as per Rouse and Pleijel [12]

120. Peristomium ring number: 0 – one ring; 1 – two rings

121. Longitudinal muscle bands

122. Circular muscles

123. Circular muscles forming complete outer layer, coded according to [52]. New character.

124. Buccal organ: 0 – absent/occluded; 1 – present

125. Buccal organ position: 0 – axial; 1 – ventral; 2 – dorsal

126. Buccal organ hypertrophied/muscularised

127. Radula. Coded as present in *Wiwaxia* [53] and all molluscs except bivalves.

128. Jaws. Although jaws among aciculate polychaetes may not be homologous, we followed [54] and coded their presence as a single state.

129. Jaw type: 0 – dorso-ventral (as in scaleworms); 1 – lateral (phyllodocidans with paired lateral jaws, as in *Alitta*); 2 – trepan (as in *Syllis*); 3 – terminal ring (as in Glyceriformia); 4 – with mandibles and maxillae (as in Eunicida)

130. Venom gland

131. Number of jaw elements: 0 – unequal; 1 – equal. Coded as equal for phyllodocidan familes with jaws and unequal for certain eunicidans [26]. New character.

132. Element size asymmetry, coded from [26]. New character.

133. Right maxilla III element, coded from [26]. New character.

134. Eunicidan jaw type: 0 – ctenognath; 1 – symmetrognath; 2 – prionognath; 3 – labidognath. The symmetrognath rather than labidognath condition is coded for Lumbrineridae [26]. New character.

135. Calcified mandibles, coded from [26]. New character.

136. Dorsal carriers, coded from [26]. New character.

137. Ventral carrier, coded from [26]. New character.

138. Maxilla I articulation, coded from [26]. New character.

139. Proboscis terminal papillae, coded from family descriptions in [12]. New character.

140. Proventricle

141. Dorsolateral folds

142. Elytra

143. Branchiae

144. Branchial type: 0 – parapodial; 1 – dorsal

145. Parapodial gills interramal. Interramal gills restricted to *Canadia, Kenostrychus* and extant *Nephtys* [55].

146. Dorsal gills anterior only

147. Nuchal organs

148. Nuchal organ structure: 0 - pits/grooves 1 - posterior projections 2 - caruncle 3 - internalized.

149. Closed circulation

150. Heart body

151. Respiratory pigment: 0 – haemoglobin; 1 – chlorocruorin; 2 – hemerythrin. Coded from [12]. Presence of hemerythrin in *Magelona* coded as per Mangum [56].

152. Rugose epidermis. Present in Capitellidae, Scalibregmatidae, Opheliidae, Arenicolidae [12, 57] and *Urechis* [58].

153. Epidermal papillae

154. Peristomial cirri. Present in *Eunice* and *Nothria* [22].

155. Chaetal inversion

156. Cilliophagocytal organ

157. Gular membrane

158. Tetraneury. Coded as present in Mollusca and Amphinomidae [59]. New character.

159. Eyes. Presence of eyes and multicellular eyes (160) coded following [5, 60, 61].

160. Multicellular adult eyes

161. Lophophore, coded as present in Brachiopoda.

162. Ventral locomotory cilia, coded as present in molluscs, the ciliary gliding “archiannelids” and *Ophelia* [3].

163. Ciliated groove

164. Muscular foot, present in molluscs and *Wiwaxia* [62].

165. Duogland system

166. Ventral nervous system

167. Nervous system position: 0 – subepidermal; 1 – intraepidermal

168. Corpora pedunculata

169. Collagenous cuticle. Coded as present in annelids (including Myzostomidae), sipunculans and echiurans [63]. Also coded as present in fossil taxa that preserve cuticle, such as those from the Burgess Shale. New character.

170. Caudal appendage, coded as present in *Golfingia* [13]

171. Perioral tentacles [13]

172. Perioral tentacle position: 0 – encircling mouth; 1 – tentacles arranged in an arc around the nuchal organ [13]

173. Helical coiling of gut. Present in all extant sipunculans in matrix but absent in the Chengjiang taxa [64].

174. Body with trunk and introvert, coded as present in sipunculans.

175. Introvert longer than trunk [13].

176. Cuticular hooks [13]

177. Cuticular hooks in rings [13]

178. Scattered cuticular hooks [13]

179. Spiral cleavage, coded from [65]

180. Circumoral nerve ring, coded as per [2].

181. Protrusible retractable chaetae, coded as present for annelids as per [65].

182. Pedicle, present in brachiopods

183. Mantle cavity, present in chitons and gastropods

184. Shell, present in *Halkieria*, chitons and gastropods.

185. Shell type: 0 – anterior and posterior with or without intermediate plates (for *Halkieria* and chitons); 1 – dorsal and ventral (brachiopods); 2 – dextrally coiled (gastropods)

186. Unpigmented cilliary ocelli

187. Light sensitive statocysts

188. Nuchal commisure

189. Cerebral commissures

190. Commissural ganglion

191. Dorsal ganglia

192. Ventral glandular area on anterior segments

Coding of fossil taxa

Fossils were coded based on the published literature and observations of specimens held at the Royal Ontario Museum (ROM) and and the United States National Museum of Natural History, Smithsonian Institution, for taxa from the Burgess Shale and the ROM for specimens from Mazon Creek.

References for character states of fossil taxa

Wiwaxia [8, 38, 51, 53, 62]
Halkieria [46, 66]
Archaeogolfingia [64]
Cambrosipunculus [64]
Arkonips [67]
Burgessochaeta [6, 8, 68]
Canadia [6, 8, 68]
Dryptoscolex [69, 70]
Esconites [48]
Fossundecima [69, 70]
Kenostrychus [55]

Mazopherusa [49]

Phragmochaeta [6, 71]

ROM62972 [6]

Supplementary references

[1] Rouse, G.W. & Fauchald, K. 1997 Cladistics and polychaetes. *Zoologica Scripta* **26**, 139-204.
[2] Rouse, G.W. 1999 Trochophore concepts: ciliary bands and the evolution of larvae in spiralian Metazoa. *Biological Journal of the Linnean Society* **66**, 411-464.
[3] Zrzavy, J., Riha, P., Pialek, L. & Janouskovec, J. 2009 Phylogeny of Annelida (Lophotrochozoa): total-evidence analysis of morphology and six genes. *BMC Evolutionary Biology* **9**. (doi:10.1186/1471-2148-9-189).
[4] Struck, T., Paul, C., Hill, N., Hartmann, S., Hosel, C., Kube, M., Lieb, B., Meyer, A., Tiedemann, R., Purschke, G., et al. 2011 Phylogenomic analyses unravel annelid evolution. *Nature* **471**, 95-113. (doi:10.1038/nature09864).
[5] Weigert, A., Helm, C., Meyer, M., Nickel, B., Arendt, D., Hausdorf, B., Santos, S.R., Halanych, K.M., Purschke, G. & Bleidorn, C. 2014 Illuminating the base of the annelid tree using transcriptomics. *Molecular Biology and Evolution* **31**, 1391-1401.
[6] Parry, L., Vinther, J. & Edgecombe, G.D. 2015 Cambrian stem-group annelids and a metameric origin of the annelid head. *Biology Letters* **11**, 20150763.
[7] Vinther, J., Eibye-Jacobsen, D. & Harper, D.A. 2011 An Early Cambrian stem polychaete with pygidial cirri. *Biology Letters* **7**, 929-932. (doi:10.1098/rsbl.2011.0592).
[8] Eibye-Jacobsen, D. 2004 A reevaluation of *Wiwaxia* and the polychaetes of the Burgess Shale. *Lethaia* **37**, 317-335. (doi:10.1080/00241160410002027).
[9] Orrhage, L. & Müller, M.C. 2005 Morphology of the nervous system of Polychaeta (Annelida). In *Morphology, Molecules, Evolution and Phylogeny in Polychaeta and Related Taxa* (pp. 79-111, Springer.
[10] Purschke, G. 2015 24 Annelida: Basal groups and Pleistoannelida. . *Structure and evolution of invertebrate nervous systems*, 768.
[11] Müller, M. & Westheide, W. 2002 Comparative analysis of the nervous systems in presumptive progenetic dinophilid and dorvilleid polychaetes (Annelida) by immunohistochemistry and cLSM. *Acta Zoologica* **83**, 33-48.
[12] Rouse, G. & Pleijel, F. 2001 *Polychaetes*, Oxford University Press; 354 p.
[13] Schulze, A., Cutler, E.B. & Giribet, G. 2007 Phylogeny of sipunculan worms: a combined analysis of four gene regions and morphology. *Molecular phylogenetics and evolution* **42**, 171-192.
[14] Merz, R.A. & Woodin, S.A. 2006 Polychaete chaetae: Function, fossils, and phylogeny. *Integrative and Comparative Biology* **46**, 481-496.
[15] Orrhage, L. 1993 On the microanatomy of the cephalic nervous system of Nereidae (Polychaeta), with a preliminary discussion of some earlier theories on the segmentation of the polychaete brain. *Acta Zoologica* **74**, 145-172.
[16] Fauchald, K. 1977 The polychaete worms; definitions and keys to the orders, families and genera.
[17] Beesley, P.L., Ross, G.J. & Glasby, C.J. 2000 *Polychaetes & allies: the southern synthesis*, CSIRO publishing.

[18] Zhadan, A., Vortsepneva, E. & Tzetlin, A. 2014 Three-dimensional reconstruction of the musculature of *Cossura pygodactylata* Jones, 1956 (Annelida: Cossuridae). *Zoologischer Anzeiger* **253**, 181-191.
[19] Eriksson, M.E., Hints, O., Paxton, H. & Tonarová, P. 2013 Ordovician and Silurian polychaete diversity and biogeography. *Geological Society, London, Memoirs* **38**, 265-272.
[20] Orrhage, L. 1995 On the innervation and homologues of the anterior end appendages of the Eunicea (Polychaeta), with a tentative outline of the fundamental constitution of the cephalic nervous system of the polychaetes. *Acta Zoologica* **76**, 229-248.
[21] Borda, E., Yáñez ‐ Rivera, B., Ochoa, G.M., Kudenov, J.D., Sanchez ‐ Ortiz, C., Schulze, A. & Rouse, G.W. 2015 Revamping Amphinomidae (Annelida: Amphinomida), with the inclusion of *Notopygos*. *Zoologica Scripta* **44**, 324-333.
[22] Paxton, H. 1986 Generic revision and relationships of the family Onuphidae (Annelida: Polychaeta). *Records of the Australian Museum* **38**, 1-74.
[23] Orrhage, L. 1991 On the innervation and homologues of the cephalic appendages of the Aphroditacea (Polychaeta). *Acta Zoologica* **72**, 233-246.
[24] Worsaae, K., Nygren, A., Rouse, G.W., Giribet, G., Persson, J., Sundberg, P. & Pleijel, F. 2005 Phylogenetic position of Nerillidae and Aberranta (Polychaeta, Annelida), analysed by direct optimization of combined molecular and morphological data. *Zoologica Scripta* **34**, 313-328.
[25] Pernet, B., Harris, L.H. & Schroeder, P. 2015 Development and Larval Feeding in the Capitellid Annelid *Notomastus cf. tenuis*. *The Biological Bulletin* **228**, 25-38.
[26] Paxton, H., Maciolek, N. & Blake, J. 2009 Phylogeny of Eunicida (Annelida) based on morphology of jaws. *Proceedings of the 9th International Polychaete Conference* **2**, 241-264.
[27] Capa, M., Parapar, J. & Hutchings, P. 2012 Phylogeny of Oweniidae (Polychaeta) based on morphological data and taxonomic revision of Australian fauna. *Zoological Journal of the Linnean Society* **166**, 236-278.
[28] Cunningham, J.A., Vargas, K., Pengju, L., Belivanova, V., Marone, F., Martínez-Pérez, C., Guizar-Sicairos, M., Holler, M., Bengtson, S. & Donoghue, P.C. 2015 Critical appraisal of tubular putative eumetazoans from the Ediacaran Weng'an Doushantuo biota. In *Proc. R. Soc. B* (p. 20151169, The Royal Society.
[29] Martínez, A., Di Domenico, M. & Worsaae, K. 2013 Evolution of cave Axiokebuita and Speleobregma (Scalibregmatidae, Annelida). *Zoologica Scripta* **42**, 623-636.
[30] Hausen, H. 2005 Chaetae and chaetogenesis in polychaetes (Annelida). *Hydrobiologia* **535**, 37-52.
[31] Helm, C. & Capa, M. 2015 Comparative analyses of morphological characters in Sphaerodoridae and allies (Annelida) revealed by an integrative microscopical approach. *Frontiers in Marine Science* **1**, 82.
[32] Filippova, A., Purschke, G., Tzetlin, A.B. & Müller, M. 2010 Musculature in polychaetes: comparison of *Myrianida prolifera* (Syllidae) and *Sphaerodoropsis* sp.(Sphaerodoridae). *Invertebrate Biology* **129**, 184-198.
[33] Hessling, R. 2002 Metameric organisation of the nervous system in developmental stages of *Urechis caupo* (Echiura) and its phylogenetic implications. *Zoomorphology* **121**, 221-234.
[34] Kristof, A., Wollesen, T. & Wanninger, A. 2008 Segmental mode of neural patterning in Sipuncula. *Current Biology* **18**, 1129-1132.
[35] Bartolomaeus, T. & Quast, B. 2005 Structure and development of nephridia in Annelida and related taxa. *Hydrobiologia* **535**, 139-165.
[36] Nielsen, C. 1991 The development of the brachiopod *Crania* (Neocrania) anomala (OF Müller) and its phylogenetic significance. *Acta Zoologica* **72**, 7-28.
[37] Young, C.M. 2006 Atlas of marine invertebrate larvae.

[38] Zhang, Z., Smith, M.R. & Shu, D. 2015 New reconstruction of the *Wiwaxia* scleritome, with data from Chengjiang juveniles. *Scientific reports* **5**.
[39] Tilic, E., Lehrke, J. & Bartolomaeus, T. 2015 Homology and evolution of the chaetae in Echiura (annelida). *PloS one* **10**, e0120002.
[40] Hessling, R. & Westheide, W. 2002 Are Echiura derived from a segmented ancestor? Immunohistochemical analysis of the nervous system in developmental stages of Bonellia viridis. *Journal of Morphology* **252**, 100-113.
[41] Wanninger, A., Koop, D., Bromham, L., Noonan, E. & Degnan, B.M. 2005 Nervous and muscle system development in *Phascolion strombus* (Sipuncula). *Development Genes and Evolution* **215**, 509-518.
[42] Goto, R., Okamoto, T., Ishikawa, H., Hamamura, Y. & Kato, M. 2013 Molecular phylogeny of echiuran worms (phylum: Annelida) reveals evolutionary pattern of feeding mode and sexual dimorphism. *PloS one* **8**, e56809.
[43] Rouse, G. 2000 Polychaetes have evolved feeding larvae numerous times. *Bulletin of marine Science* **67**, 391-409.
[44] Rouse, G.W. 2000 The epitome of hand waving? Larval feeding and hypotheses of metazoan phylogeny. *Evolution & development* **2**, 222-233.
[45] Bartolomaeus, T. 1999 Structure, function and development of segmental organs in Annelida. *Hydrobiologia* **402**, 21-37.
[46] Conway Morris, S. & Peel, J.S. 1995 Articulated halkieriids from the Lower Cambrian of North Greenland and their role in early protostome evolution. *Philosophical Transactions of the Royal Society of London B: Biological Sciences* **347**, 305-358.
[47] Vinther, J. 2015 The origins of molluscs. *Palaeontology* **58**, 19-34.
[48] Thompson, I. & Johnson, R.G. 1977 *New fossil polychaete from Essex, Illinois*, Field Museum of Natural History.
[49] Hay, A.A. 2002 Flabelligerida from the Francis Creek shale of Illinois. *Journal of Paleontology* **76**, 764-766.
[50] Bouché, M.-L., Biagianti-Risbourg, S. & Vernet, G. 1999 A light and scanning electron microscope study of the morphology of the chaetae of *Tubifex tubifex* in a non-polluted medium. *Hydrobiologia* **411**, 39-44.
[51] Butterfield, N.J. 1990 A reassessment of the enigmatic Burgess Shale fossil *Wiwaxia corrugata* (Matthew) and its relationship to the polychaete *Canadia spinosa* Walcott. *Paleobiology* **16**, 287-303.
[52] Purschke, G. & Müller, M. 2006 Evolution of body wall musculature. *Integrative and comparative biology* **46**, 497-507.
[53] Smith, M.R. 2012 Mouthparts of the Burgess Shale fossils *Odontogriphus* and *Wiwaxia*: implications for the ancestral molluscan radula. *Proceedings of the Royal Society B: Biological Sciences*, rspb20121577.
[54] Eibye-Jacobsen, D. & Vinther, J. 2012 Reconstructing the ancestral annelid. *Journal of Zoological Systematics and Evolutionary Research* **50**, 85-87. (doi:10.1111/j.1439-0469.2011.00651.x).
[55] Sutton, M.D., Briggs, D.E., Siveter, D.J. & Siveter, D.J. 2001 A three-dimensionally preserved fossil polychaete worm from the Silurian of Herefordshire, England. *Proceedings of the Royal Society B: Biological Sciences*, 2355-2363.
[56] Mangum, C.P. 1992 Physiological function of the hemerythrins. In *Blood and Tissue Oxygen Carriers* (pp. 173-192, Springer.
[57] Fauchald, K. & Rouse, G. 1997 Polychaete systematics: past and present. *Zoologica Scripta* **26**, 71-138.
[58] Menon, J.G. & Arp, A.J. 1993 The integument of the marine echiuran worm *Urechis caupo*. *The Biological Bulletin* **185**, 440-454.
[59] Marsden, J.R. 1966 Segmental musculature and locomotion in Hermodice carunculata (Polychaeta: Amphinomidae). *Journal of Morphology* **119**, 259-276.

[60] Suschenko, D. & Purschke, G. 2009 Ultrastructure of pigmented adult eyes in errant polychaetes (Annelida): implications for annelid evolution. *Zoomorphology* **128**, 75-96.
[61] Purschke, G., Arendt, D., Hausen, H. & Müller, M.C. 2006 Photoreceptor cells and eyes in Annelida. *Arthropod structure & development* **35**, 211-230.
[62] Smith, M.R. 2014 Ontogeny, morphology and taxonomy of the soft‐bodied Cambrian 'mollusc' *Wiwaxia*. *Palaeontology* **57**, 215-229.
[63] Hausen, H. 2005 Comparative structure of the epidermis in polychaetes (Annelida). *Hydrobiologia* **535**, 25-35.
[64] Huang, D.Y., Chen, J.Y., Vannier, J. & Salinas, J.I.S. 2004 Early Cambrian sipunculan worms from southwest China. *Proceedings of the Royal Society B-Biological Sciences* **271**, 1671-1676. (doi:10.1098/rspb.2004.2774).
[65] Peterson, K.J. & Eernisse, D.J. 2001 Animal phylogeny and the ancestry of bilaterians: inferences from morphology and 18S rDNA gene sequences. *Evolution & development* **3**, 170-205.
[66] Vinther, J. & Nielsen, C. 2005 The early Cambrian *Halkieria* is a mollusc. *Zoologica Scripta* **34**, 81-89.
[67] Farrell, U. & Briggs, D. 2007 A pyritized polychaete from the Devonian of Ontario. *Proceedings of the Royal Society B-Biological Sciences* **274**, 499-504. (doi:10.1098/rspb.2006.0063).
[68] Conway Morris, S. 1979 Middle Cambrian polychaetes from the Burgess shale of British Columbia. *Philosophical Transactions of the Royal Society of London. Series B, Biological Sciences* **285**, 227-274.
[69] Fitzhugh, K., Sroka, S., Kruty, M., Henderson, A. & Hay, A. 1997 Polychaete worms. *Richardson's Guide to the Fossil Fauna of Mazon creek*, 64-83.
[70] Thompson, I. 1979 Errant polychaetes (Annelida) from the Pennsylvanian Essex fauna of northern Illinois. *Palaeontographica Abteilung A* **163**, 169-199.
[71] Conway Morris, S. & Peel, J. 2008 The earliest annelids: Lower Cambrian polychaetes from the Sirius Passet Lagerstatte, Peary Land, North Greenland. *Acta Palaeontologica Polonica* **53**, 135-146.

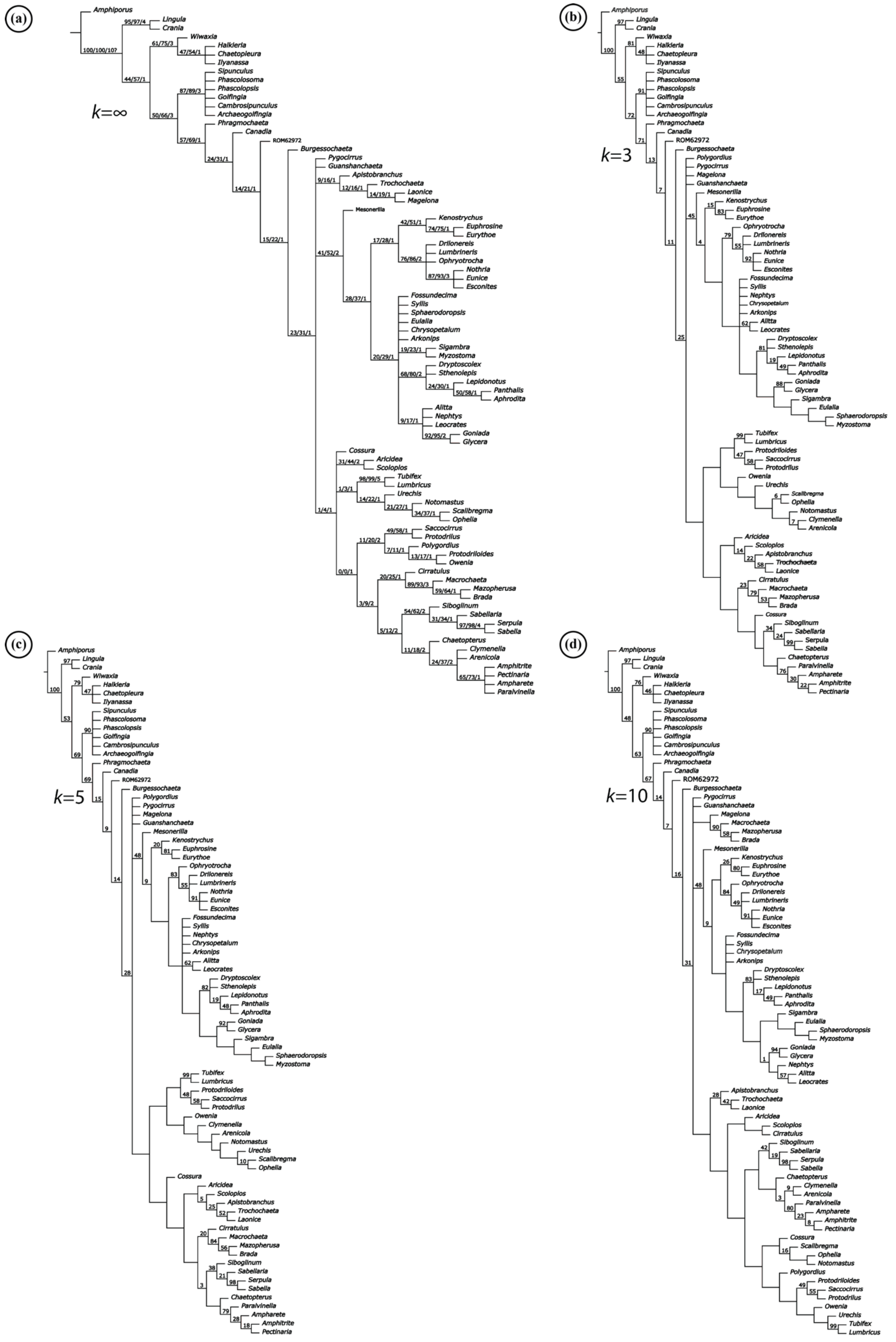

Supplementary Figure 1. Results of parsimony analyses of discrete character data; (*a*) Equal character weighting, numbers at nodes are bootstrap/jackknife/Bremer support values; (*b*) Implied weighting (*k*=3), (c) Implied weighting (*k*=5); (*d*) Implied weighting (*k*=10). Numbers at nodes of implied weighting are support values from symmetric resampling.

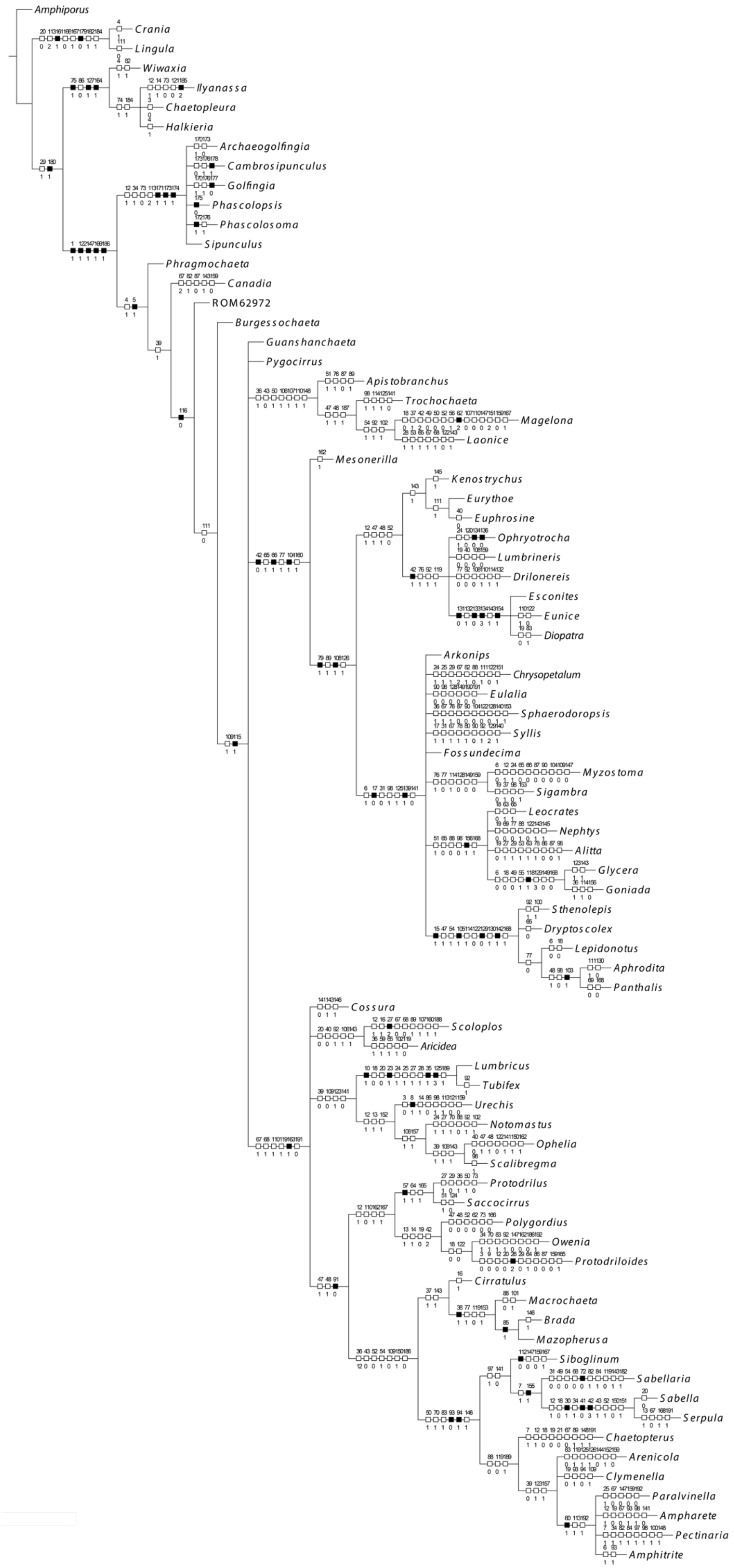

Supplementary figure 2. Apomorphies optimized on an equal weights parsimony tree. Only unambiguous changes are shown. Numbers above and below branches are character numbers and states respectively. Black boxes are non-homoplastic characters and white boxes are homoplastic changes. Unsupported nodes were hard collapsed following character optimization.

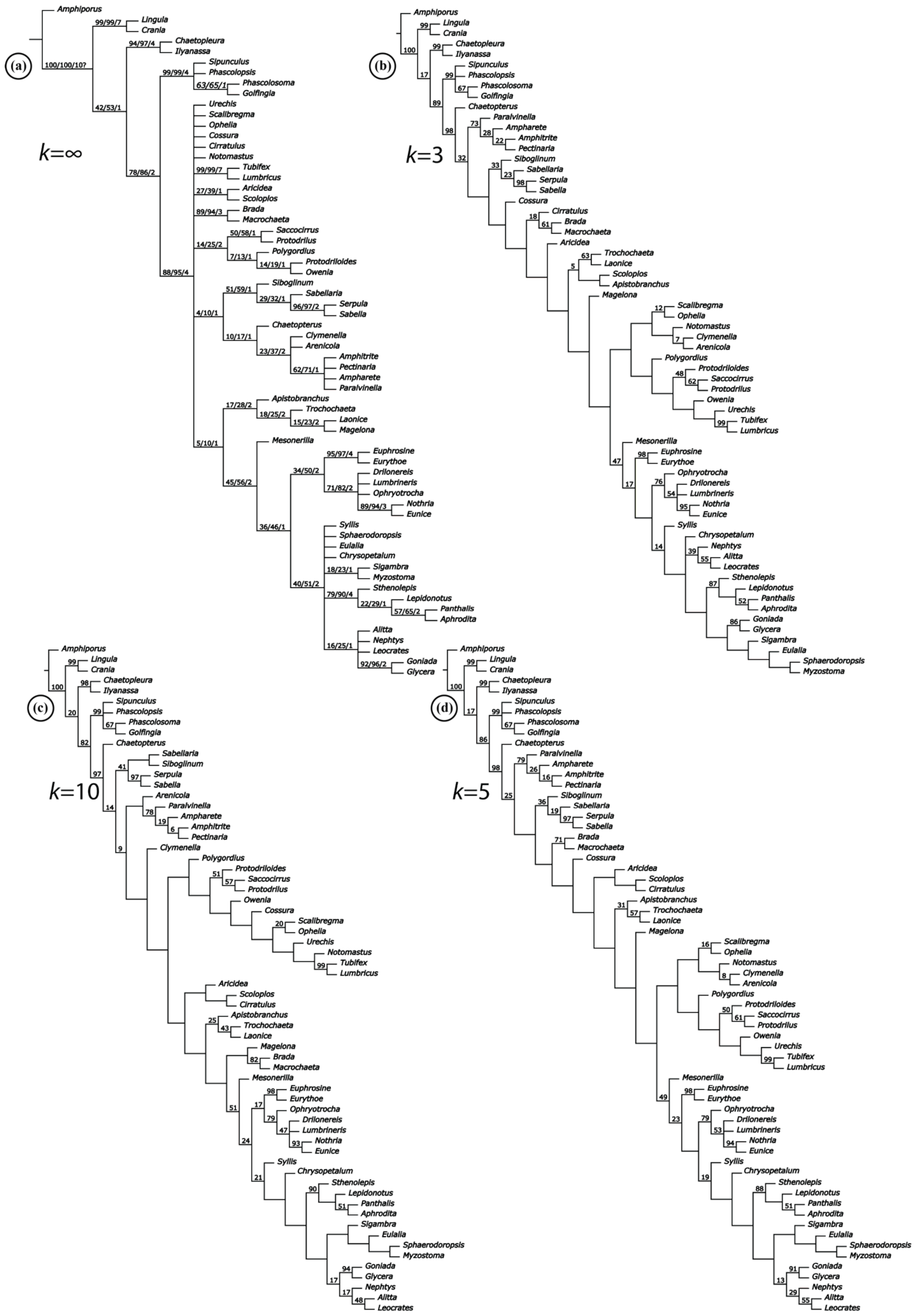

Supplementary Figure 3. Parsimony analyses of discrete character data excluding fossils. (*a*) Equal character weighting, numbers at nodes are bootstrap/jackknife/Bremer support values. (*b*) Implied weighting (*k*=3), (*c*) Implied weighting (*k*=5); (*d*) Implied weighting (*k*=10). Numbers at nodes of implied weighting are support values from symmetric resampling.

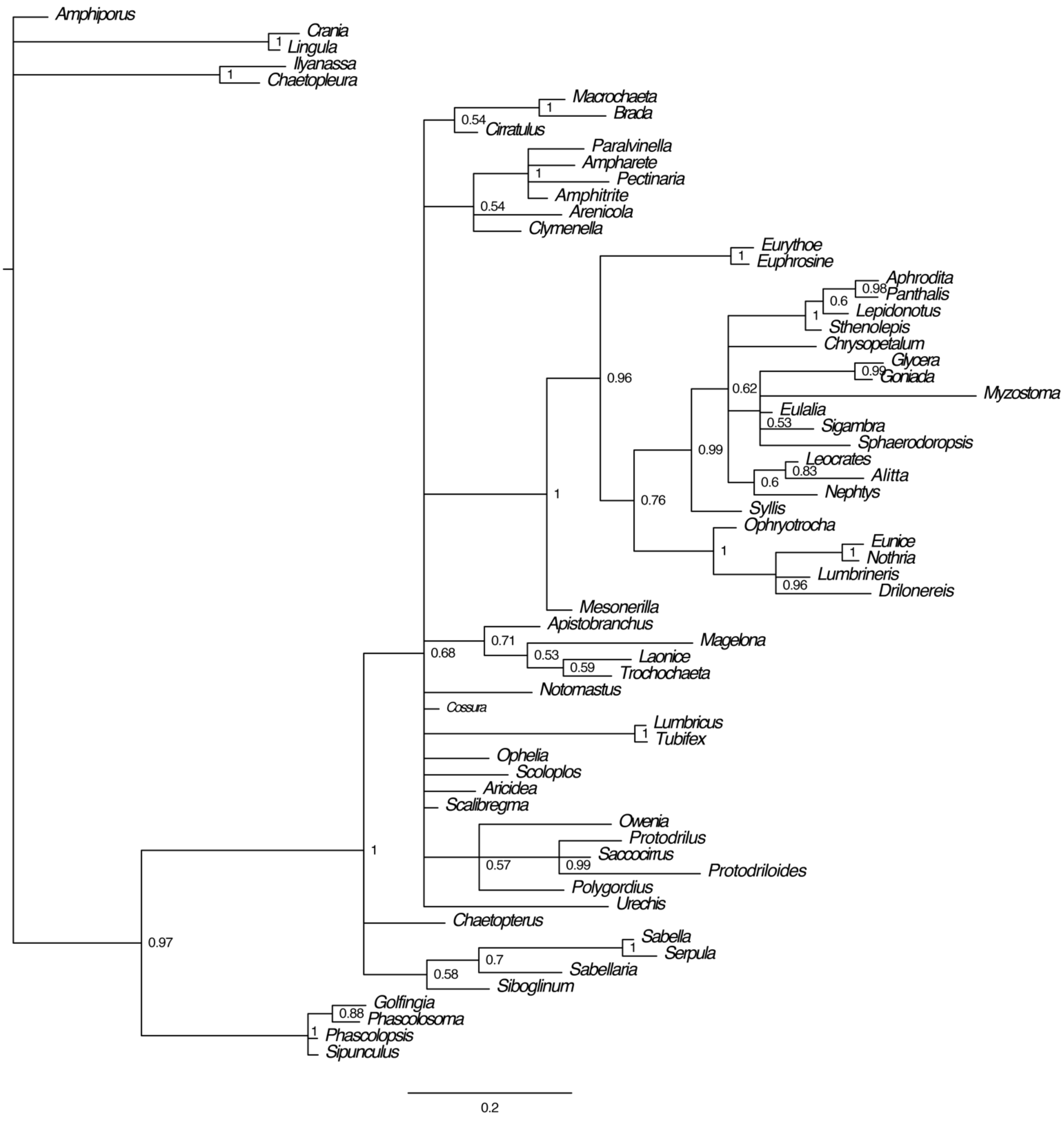

Supplementary Figure 4. Majority rule consensus tree of Bayesian analysis using the *mkv* model excluding fossil taxa. Numbers at nodes are posterior probabilities.

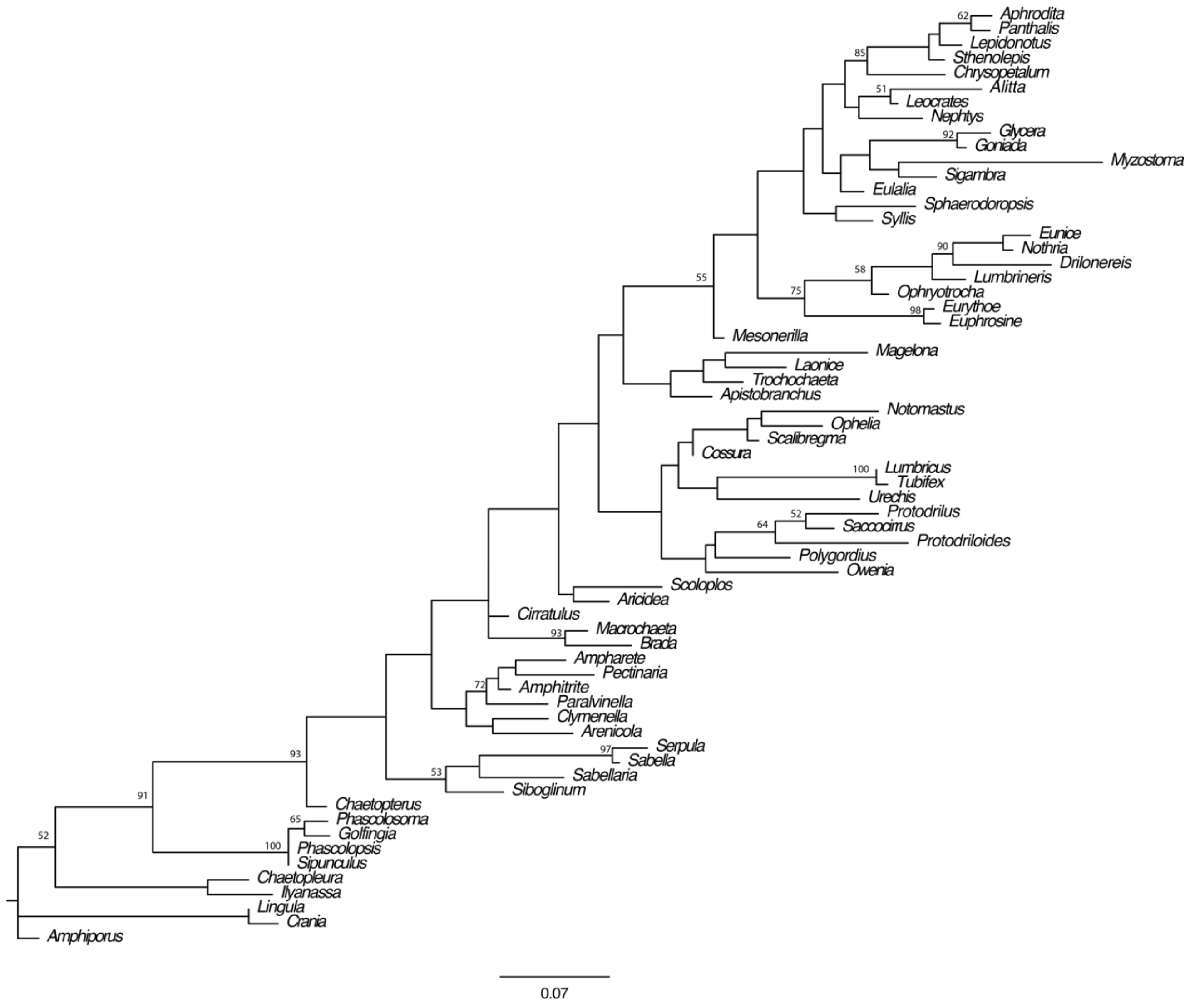

Supplementary Figure 5. Maximum likelihood phylogram with fossil taxa excluded. Numbers at nodes are support values from 1000 bootstrap replicates.

	ew	iwgk3	ml	BI	%missing
Canadia	5	5	5	5	40.63
Cambrosipunculus	4	4	4	4	41.15
Burgessochaeta	7	7	7	7	42.19
Archaeogolfingia	4	4	4	4	42.19
Phragmochaeta	4	4	4	4	43.75
ROM62972	6	6	6	6	44.79
Pygocirrus	8	8	8	8	47.40
Wiwaxia	3	3	3	3	48.44
Guanshanchaeta	8	8	9	8	49.48
Esconites	13	14	16	14	52.60
Dryptoscolex	12	14	18	12	54.17
Fossundecima	11	12	17	11	55.21
Mazopherusa	14	14	16	11	55.73
Kenostrychus	12	11	12	10	56.25
Arkonips	11	12	16	11	63.02
Halkieria	4	4	4	4	63.02

Supplementary Table 1. Number of nodes from the root for equal weights (ew), implied weights *k*=3 (iwgk3), maximum likelihood (ml) and Bayesian inference (BI).

Optimality criterion	Pearson's r	P	Spearman's rho	P
ew	0.57	0.0225	0.54	0.0296
iwgk3	0.57	0.0209	0.53	0.0336
ML	0.61	0.0123	0.54	0.0297
BI	0.54	0.0308	0.54	0.0308

Supplementary Table 2. Pearson's r and spearman's correlation coefficients and P values for number of nodes from root versus percent missing data.

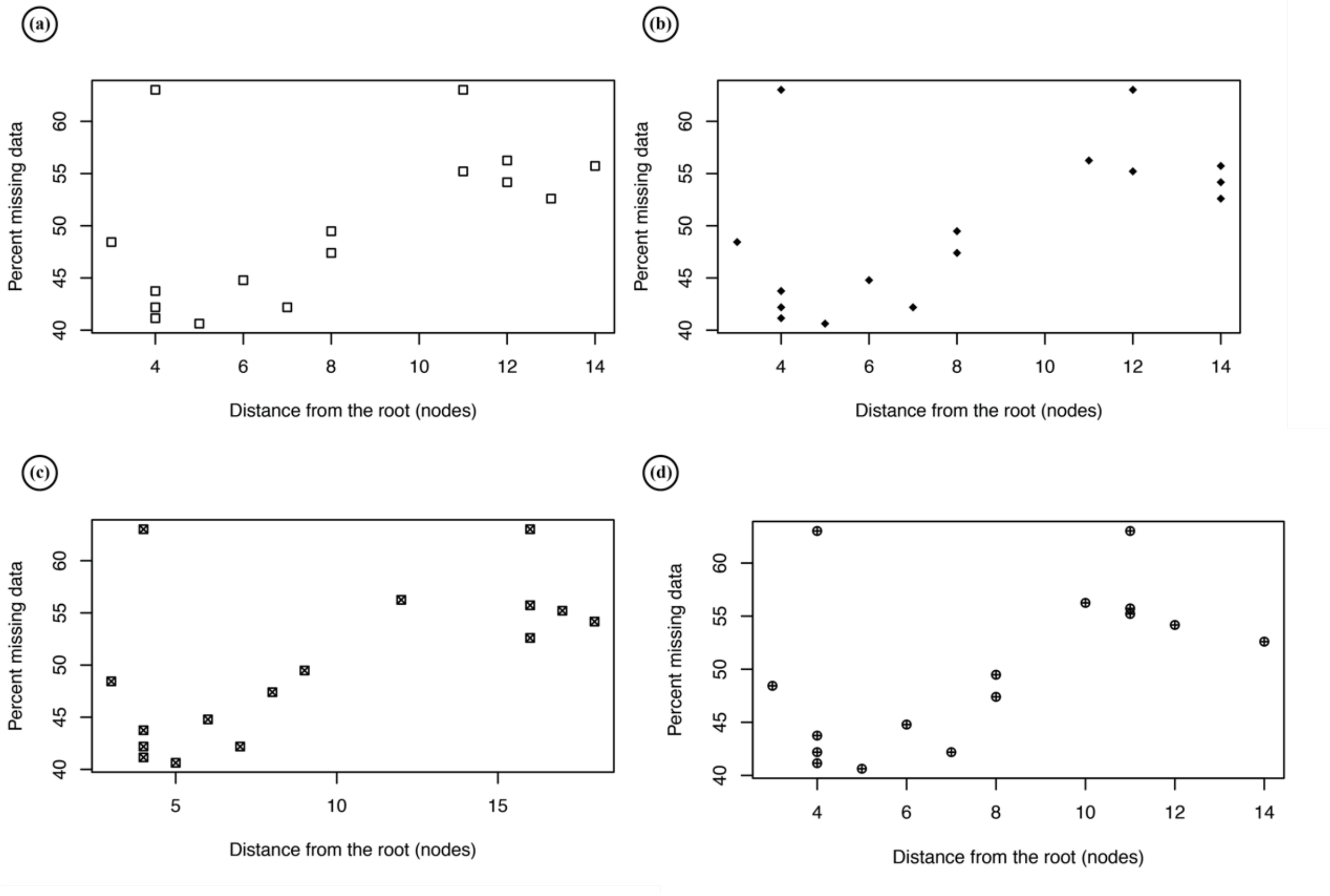

Supplementary Figure 5. Plots of percent missing data versus distance from the root. (*a*) Equal weights; (*b*) Implied weights *k*=3; (*c*) Maximum likelihood; (*d*) Bayesian inference.
